# Analysing quantification differences in key brain regions for brain perfusion SPECT scans in the diagnosis of dementia, when using different control databases

**DOI:** 10.1002/alz70856_102650

**Published:** 2025-12-24

**Authors:** Thomas Gee, Gemma Lewis, Christopher Kipps, Sofia Michopoulou

**Affiliations:** ^1^ University Hospital Southampton NHS Foundation Trust, Southampton, United Kingdom

## Abstract

**Background:**

A key aspect of the quantification of brain perfusion SPECT scans is the control database. Collecting a location specific control database is a time consuming task, due to the number of studies required to make a suitable database, and the need to know how the patient progresses after their scan. Because of this, commercial databases could provide centres with the means to efficiently perform quantification of brain perfusion SPECT scans. This study will investigate the brain regions that provide significantly different quantification results when using a commercial over a local control database.

**Method:**

MIMNeuro was used to process 35 SPECT scans, using two different control databases: one constructed at University Hospital Southampton containing 31 controls, and a commercially available database provided by MIMNeuro containing 91 controls. MIMNeuro produces z‐scores for key brain regions involved in neurodegeneration, and the results from processing with each control database were compared to determine which regions were most affected. Thresholds of 1.5 and 2.5 STDs were used to determine whether disease is moderate or severe, respectively. A two‐sample t‐test was then used to assess region significance across the dataset.

**Result:**

Initial analysis of the correlation between quantification using both methods shows the right side of the brain has stronger correlation than the left. Regions such as the Temporals, and Precuneus, which are typically associated with Alzheimer's disease, show repeated significant differences (suggested to be >20% change in z‐score) when processing with each database. A difference in z‐score of this size is likely to push a region into a different disease threshold, when near a threshold. Within the Precuneus, the commercial database indicates higher levels of disease than when using a localised database, however, the Temporals show the opposite. Further review of disease progression, after these scans, would highlight which method is most accurate.

**Conclusion:**

Reporting of the t‐test results will identify whether the differences between the two databases impacts diagnosis. While a localised control database likely provides more accurate representation of the population receiving the scan, a commercial database is a viable option for those who don’t have the capacity to collect this data.

